# Biochemical Properties of a New Polysaccharide Lyase Family 25 Ulvan Lyase TsUly25B from Marine Bacterium *Thalassomonas* sp. LD5

**DOI:** 10.3390/md20030168

**Published:** 2022-02-25

**Authors:** Danni Wang, Yujiao Li, Lu Han, Chengying Yin, Yongqing Fu, Qi Zhang, Xia Zhao, Guoyun Li, Feng Han, Wengong Yu

**Affiliations:** 1 School of Medicine and Pharmacy, Ocean University of China, 5 Yushan Road, Qingdao 266003, China; wdn@stu.ouc.edu.cn (D.W.); lyj6476@stu.ouc.edu.cn (Y.L.); hanlu3533@stu.ouc.edu.cn (L.H.); yinchengying@stu.ouc.edu.cn (C.Y.); fuyongqing@stu.ouc.edu.cn (Y.F.); zhqiouc@163.com (Q.Z.); zhaoxia@ouc.edu.cn (X.Z.); liguoyun@ouc.edu.cn (G.L.); 2 Laboratory for Marine Drugs and Bioproducts of Qingdao, National Laboratory for Marine Science and Technology, Qingdao 266237, China; 3 Key Laboratory of Marine Drugs, Ministry of Education, 5 Yushan Road, Qingdao 266003, China; 4 Shandong Provincial Key Laboratory of Glycoscience and Glycotechnology, Department of Science & Technology of Shandong Province, 5 Yushan Road, Qingdao 266003, China

**Keywords:** ulvan lyase, polysaccharide lyase, mode of action, salt tolerance, green tide

## Abstract

Marine macroalgae, contributing much to the bioeconomy, have inspired tremendous attention as sustainable raw materials. Ulvan, as one of the main structural components of green algae cell walls, can be degraded by ulvan lyase through the β-elimination mechanism to obtain oligosaccharides exhibiting several good physiological activities. Only a few ulvan lyases have been characterized until now. This thesis explores the properties of a new polysaccharide lyase family 25 ulvan lyase TsUly25B from the marine bacterium *Thalassomonas* sp. LD5. Its protein molecular weight was 54.54 KDa, and it was most active under the conditions of 60 °C and pH 9.0. The *K*_m_ and *k*_cat_ values were 1.01 ± 0.05 mg/mL and 10.52 ± 0.28 s^−1^, respectively. TsUly25B was salt-tolerant and NaCl can significantly improve its thermal stability. Over 80% of activity can be preserved after being incubated at 30 °C for two days when the concentration of NaCl in the solution is above 1 M, while 60% can be preserved after incubation at 40 °C for 10 h with 2 M NaCl. TsUly25B adopted an endolytic manner to degrade ulvan polysaccharides, and the main end-products were unsaturated ulvan disaccharides and tetrasaccharides. In conclusion, our research enriches the ulvan lyase library and advances the utilization of ulvan lyases in further fundamental research as well as ulvan oligosaccharides production.

## 1. Introduction

Marine seaweeds contribute much to photosynthesis on Earth. Marine algae, including red algae, brown algae, and green algae, have huge biomass and play a key role in marine ecosystems. As renewable energy, seaweeds with huge aquatic biomass and rapid growth velocity are getting more and more attention [1,2,3]. Polysaccharides, the main components of their biomass, can play a structural role in providing cell rigidity. Carrageenan is one of the cell wall polysaccharides of red algae. Alginate and ulvan are the most abundant cell wall polysaccharides of brown algae and green algae, respectively. There has been substantial research undertaken on the Carrageenan, alginate, and degrading enzymes. Compared with them, only a few studies on ulvan and enzymes relating to its degradation exist. Green algae *Ulva* sp. and *Enteromorpha* sp. can grow in eutrophic waters and form “green tides”, causing serious harmful effects on the ecological environment and economy of coastal cities [4]. However, from another perspective, green algae can be regarded as a potential renewable energy source for rich ulvan in biomass which can be used as a raw material for biofuel production [5]. At present, the principal obstacle to the utilization of this valuable biomass is the lack of specific tools for degradation. Therefore, the development of related tool enzymes, such as ulvan lyases which play a role in the early stage of ulvan degradation, is of great significance.

Ulvan is an abundant marine sulfated polysaccharide extracted from the cell wall of green macroalgae, which accounts for up to 30% of the dry weight of green algae, and has been regarded as an incompletely developed new resource. Repeated disaccharide modules constitute the main chain of ulvan with occasional branches in the side chain. According to different sources of ulvan, the disaccharide units can be divided into three types including A3s (GlcA β-1,4-linked to Rha3S), B3s (IdoA α-1,4-linked to Rha3S), and U3s (Xyl β-1,4-linked to Rha3S) [6,7,8]. GlcA, IdoA, Rha3S, and Xyl represent d-glucuronic acid, l-iduronic acid, l-rhamnose 3-sulfate, and d-xylose, respectively. Ulvan has many remarkable biological properties such as antioxidant, immune regulation, anti-tumor, anti-coagulation, anti-virus, and hypolipidemic activity [9,10,11,12,13,14,15] and these excellent properties indicate the multiplicity of uses. Ulvan can be applied in various fields including food, cosmetics, biomedicine, and biomaterials [16,17,18,19].

Owing to the better water solubility and smaller molecular weight in comparison to ulvan, the physiological activities of ulvan oligosaccharides have also drawn increased attention. For example, ulvan oligosaccharides have been confirmed to have antioxidant activity, antibacterial activity, and the ability to induce oxidation bursts in dicotyledonous plant cells [20,21,22]. These excellent properties indicate the potential of valuable applications in some fields such as biomedicine.

Ulvan lyases are polysaccharide lyases capable of degrading ulvan into oligosaccharides. This kind of activity was evidenced in 1997, and the first isolated enzyme with the identified sequence was in 2011 by Collen et al. [23]. Most ulvan lyases are co-localized in the bacterial genome with other enzymes involved in the degradation of ulvan [24,25,26]. β- elimination mechanism is applied by ulvan lyase to complete the ulvan depolymerization [27]. Based on the CAZy database classification result, ulvan lyases are classified into five polysaccharide lyase (PL) families: PL24, PL25, PL28, PL37, and PL40. Only a few ulvan lyases have been characterized or structure-resolved during the 10 years since the first research. Existing literature has found that all ulvan lyases that have been characterized lead to the degradation of the substrate in an endolytic manner [23,24,28,29,30,31,32,33,34]. Their optimum temperature ranges from 30 °C to 50 °C and their optimum pH ranges from 7.5 to 9.0 [24,31,32,33,34]. The structures of two PL24 ulvan lyases, LOR_107 from *Alteromonas* sp. LOR and Uly1 from *Catenovulum maritimum* Q1, one PL25 ulvan lyase PLSV_3936 from *Pseudoalteromonas* sp. strain PLSV, and one PL28 ulvan lyase NLR48 from *Nonlabens ulvanivorans* have been resolved. PL24 and PL25 ulvan lyases adopt a seven-bladed β-propeller architecture [5,30,35], while the PL28 ulvan lyase adopts a β-jelly roll fold [28].

Herein, the ulvan lyase-encoding gene *tsuly25B* was cloned from the marine bacterium *Thalassomonas* sp. LD5 and expressed in *Escherichia coli* BL21 (DE3). As an ulvan lyase possessing the highest salt tolerance among all the characterized ulvan lyases, TsUly25B exhibited good properties in the aspects of pH tolerance, temperature stability, and substrate affinity. The observation of salt bridges near the conserved catalytic sites is also previously undiscovered. Our research not only enhances our understanding of ulvan lyases but also enriches the knowledge about the utilization of ulvan and the enzymatic production of ulvan oligosaccharides.

## 2. Results

### 2.1. Isolation and Bioinformatic Analyzing of TsUly25B

**Figure 1:**
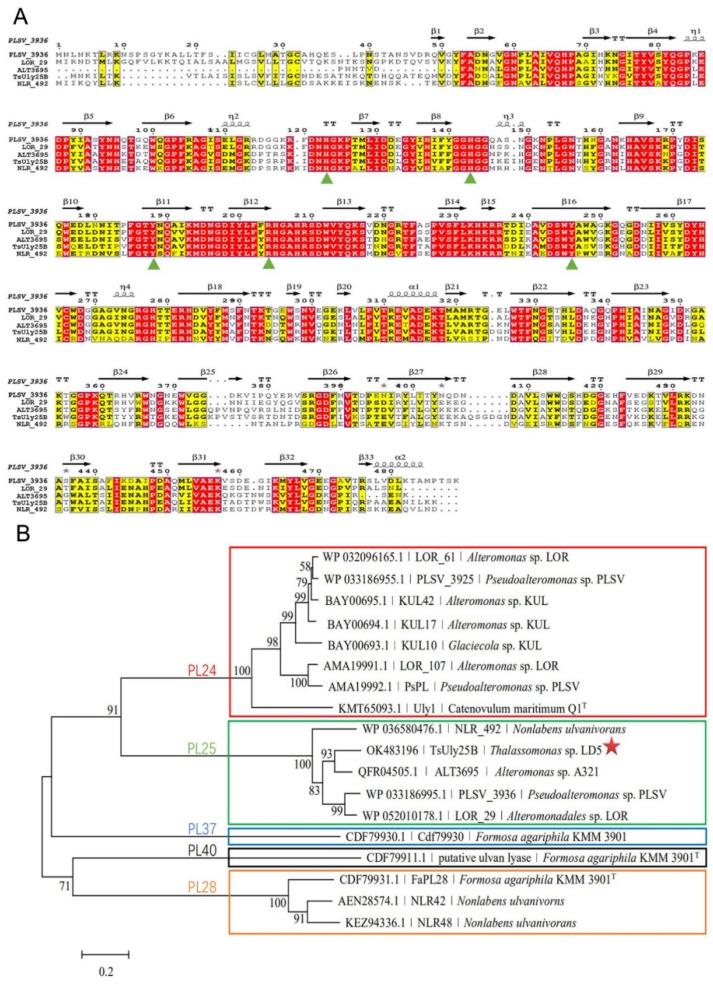
Multiple sequence alignment and phylogenetic tree analysis of TsUly25B. (**A**) Multiple sequence alignment of TsUly25B from *Thalassomonas* sp. LD5 (OK483196.1) with other characterized PL25 ulvan lyases. The secondary structure elements shown above are referenced according to PLSV_3936. PLSV_3936, from *Pseudoalteromonas* sp. PLSV (WP_033186995.1); LOR_29, from *Alteromonadales* sp. LOR (WP_052010178.1); ALT3695, from *Alteromonas* sp. A321 (QFR04505.1); NLR_492, from *N. ulvanivorans* (WP_036580476.1). The green upright solid triangle represents the conserved amino acid residues related to catalysis. (**B**) Phylogenetic tree analysis of TsUly25B with other characterized ulvan lyases.

The gene *tsuly25B* contains a 1476 bp open reading frame (ORF). The deduced protein TsUly25B is composed of 491 amino acid residues. Moreover, the Mw and pI of TsUly25B are 54.54 kDa and 6.09, respectively. The signal peptide of TsUly25B was predicted at the N-terminal (Met^1^-Asn^22^). According to the result of multiple sequence alignment, TsUly25B contains five highly conserved PL25 amino acids including His^117^, His^137^, Tyr^182^, Arg^198^, and Tyr^240^ which have been proved to be crucial to catalytic reaction in PLSV_3936 (Figure 1A). A phylogenetic tree (Figure 1B) was constructed using the amino acid sequences of TsUly25B with other characterized ulvan lyases. The homology of TsUly25B with the closest PL25 ulvan lyase which is ALT3695 [31] is 75.63%. In line with the results of homology alignment and phylogenetic analysis, TsUly25B was implied to be classified into PL25. 

**Figure 2:**
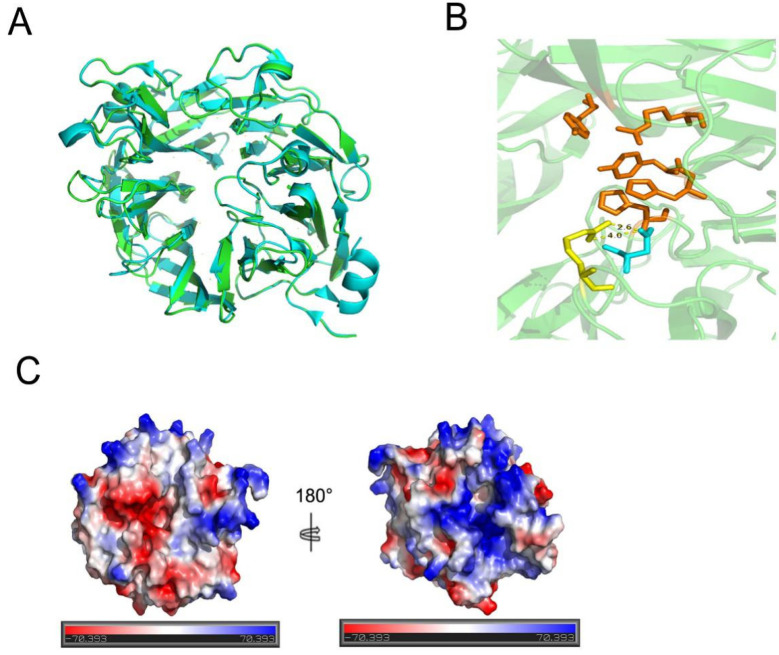
Structure modeling and analysis of TsUly25B. (**A**) Three-dimensional structure comparison of TsUly25B and PLSV_3936. TsUly25B, green; PLSV_3936 (PDB ID: 5UAM), blue. Homology modeling of TsUly25B was conducted using PLSV_3936 as the template. (**B**) Two salt bridges between Arg^112^ and Asp^115^ were found near the conserved catalytic sites (within 4 Å) in the catalytic cavity of TsUly25B. Orange sticks represent conserved catalytically related amino acid residues. Yellow sticks represent Arg^112^ and blue sticks represent Asp^115^. (**C**) Electrostatic potential distribution of TsUly25B.

PLSV_3936 (PDB ID: 5UAM) was used as a template to obtain the structure of TsUly25B through homology modeling (Figure 2A). TsUly25B showed the identity of 58.82% with PLSV_3936 (GenBank accession number: WP_033186995) and shared a highly similar structure with PLSV_3936 (Figure 2A).

### 2.2. Recombinant Expression and Purification of TsUly25B

**Figure 3:**
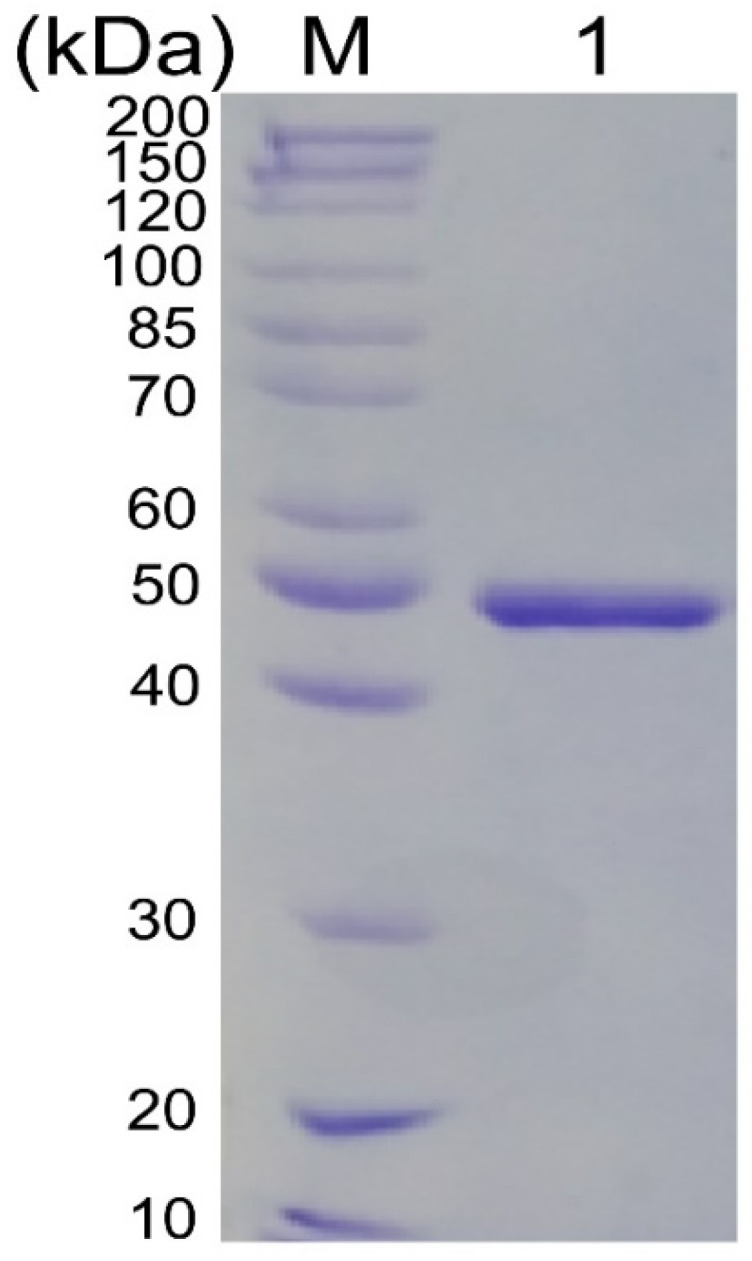
SDS-PAGE (10%, *w*/*v*) detection of purified enzyme. Lane M, protein standard marker; lane 1, purified recombinant TsUly25B.

The gene *tsuly25B* was cloned into the pET-28a (+) vector and expressed in *E. coli* BL21(DE3). The enzyme production reached 594.5 mg/L, and a majority of goal proteins were expressed in a soluble fraction. The recombinant TsUly25B was purified to homogeneity with a specific activity of 0.23 ± 0.09 U/mg. A single band showing a protein molecular weight of 50 kDa was displayed on SDS-PAGE (Figure 3), in keeping with its theoretical Mw.

### 2.3. Biochemical Characterization of Recombinant TsUly25B

**Figure 4:**
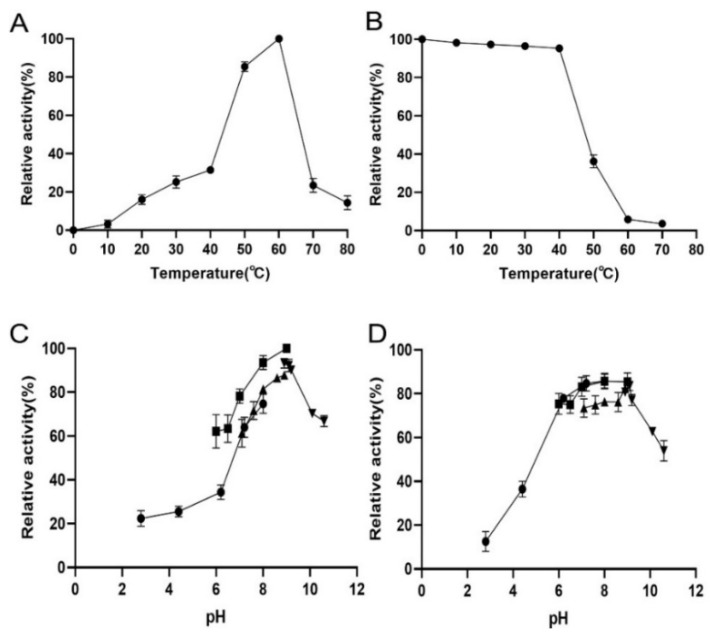
Biochemical properties of recombinant TsUly25B. (**A**) The optimal temperature of TsUly25B. The highest activity at 60 °C was set as 100%. (**B**) Thermal stability of TsUly25B. The initial activity measured at 40 °C was set as 100%. (**C**) Optimal pH of TsUly25B. (**D**) pH stability of TsUly25B. For (**C**,**D**), the solid upside-down triangle represents Glycine-NaOH (50 mM, pH 8.6–10.6). The solid upright triangle represents Tris-HCl (50 mM, pH 7.05–8.95). The solid square represents Na_2_HPO_4_-NaH_2_PO_4_ (50 mM, pH 6.0–8.0). The solid circle represents Na_2_HPO_4_ citric acid (50 mM, pH 3.0–8.0). The activity of TsUly25B at the optimal pH and temperature was defined as 100%. Experiments were conducted three times and error bars represent standard deviations.

The recombinant TsUly25B displayed the maximum activity at 60 °C (Figure 4A). According to the thermal stability results, TsUly25B kept stable when at and below 40 °C (Figure 4B). The optimal pH of TsUly25B detected at 60 °C was pH 9.0 (Figure 4C). The fact that TsUly25B kept steady at pH 6.0–10.0 (Figure 4D) indicates that it could remain stable over a broad range of pH.

**Figure 5:**
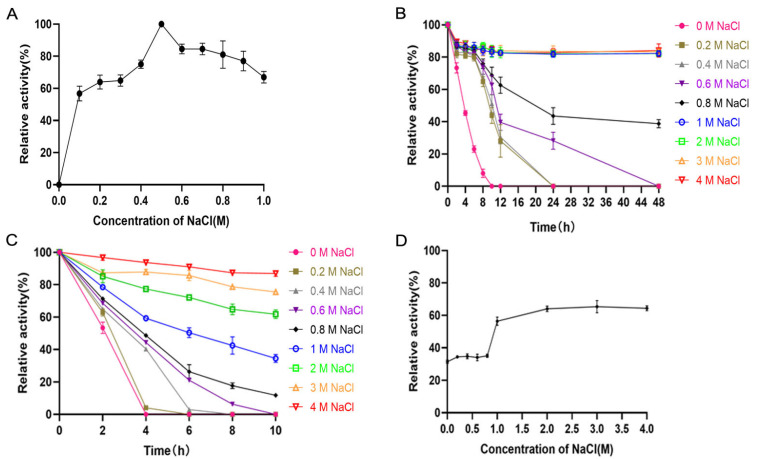
Influence of NaCl on the activity and stability of TsUly25B. (**A**) Effects of NaCl concentrations (0–1 M). The highest activity at 500 mM NaCl was set as 100%. (**B**) Influence of NaCl on the stability of TsUly25B at 30 °C. The protein was added into 20 mM PB containing a range of NaCl and incubated at 30 °C for 0, 2, 4, 6, 8, 10, 12, 24, 48 h, respectively. Then the remaining activity was confirmed at optimal temperature. The initial activity of TsUly25B at the optimal pH, temperature and NaCl concentration was defined as 100%. (**C**) Influence of NaCl on the stability of TsUly25B at 40 °C. The protein was added into 20 mM PB containing NaCl with a range of concentrations and incubated at 40 °C for 0, 2, 4, 6, 8, 10 h, respectively. Then the remaining activity was confirmed at optimal temperature. The initial activity of TsUly25B at the optimal pH, temperature and NaCl concentration was defined as 100%. (**D**) Influence of NaCl on the stability of TsUly25B at 50 °C. The protein was added into 20 mM PB containing NaCl (0 M, 0.2 M, 0.4 M, 0.6 M, 0.8 M, 1 M, 2 M, 3 M, 4 M) and incubated at 50 °C for 1 h. Then the remaining activity was confirmed at optimal temperature. The initial activity of TsUly25B at the optimal pH, temperature and NaCl concentration was defined as 100%. For (**B**,**C**), the red upside-down hollow triangle represents 4 M NaCl. The orange upright hollow triangle represents 3 M NaCl. The green hollow square represents 2 M NaCl. The blue hollow circle represents 1 M NaCl. The black solid rhombus represents 0.8 M NaCl. The purple upside-down solid triangle represents 0.6 M NaCl. The grey upright solid triangle represents 0.4 M NaCl. The brown solid square represents 0.2 M NaCl. The solid pink circle represents 0 M NaCl. PB represents Na_2_HPO_4_-NaH_2_PO_4_ buffer. Experiments were conducted three times and error bars represent standard deviations.

Recombinant TsUly25B exhibited the highest activity in the presence of 500 mM NaCl (Figure 5A). Over 80% of activity can be retained after being incubated at 30 °C for two days when the concentration of NaCl was above 1 M (Figure 5B), while 60% can be preserved at 40 °C for 10 h in the presence of 2 M NaCl (Figure 5C). After being placed at 50 °C for 1 h with 1 M NaCl, TsUly25B still maintained more than 60% of activity (Figure 5D).

### 2.4. Enzymatic Reaction Kinetics of TsUly25B

Enzymatic reaction kinetics of TsUly25B were determined using Lineweaver–Burk plots. The *V*_max_ was 0.039 ± 0.24 μmol·min^−1^·mL^−1^. The *K*_m_ and *k*_cat_ values were 1.01 ± 0.31 mg/mL and 10.52 ± 0.28 s^−1^, respectively. Its specific activity was 0.23 ± 0.09 U/mg.

### 2.5. Action Pattern and End Products of TsUly25B

**Figure 6:**
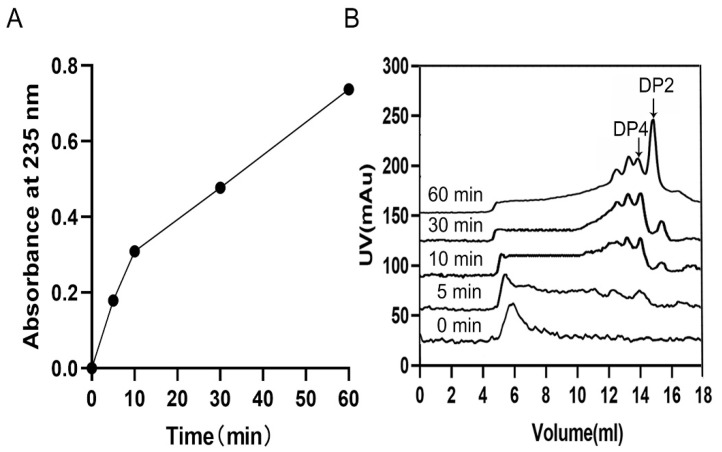
Action mode of TsUly25B. (**A**) Absorbance variation at 235 nm during the SEC analysis. The absorbance was tested at 0 min, 5 min, 10 min, 30 min, and 60 min. (**B**) Time course of ulvan degradation monitored by SEC to demonstrate the action mode. DP represents the degree of polysaccharides.

**Figure 7:**
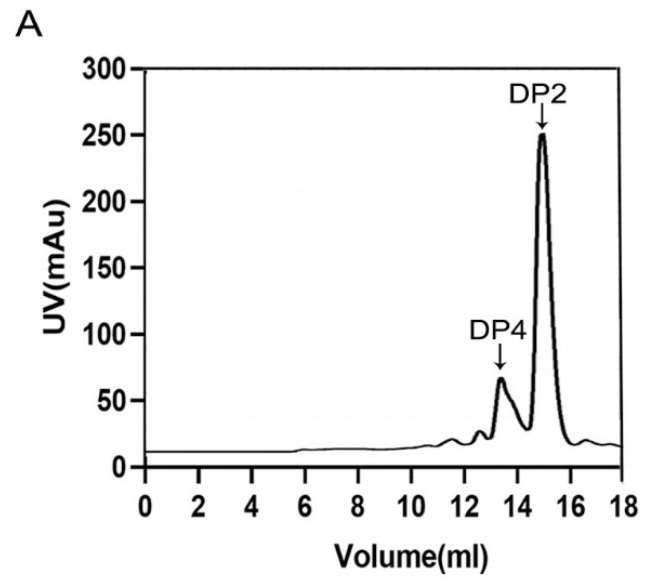

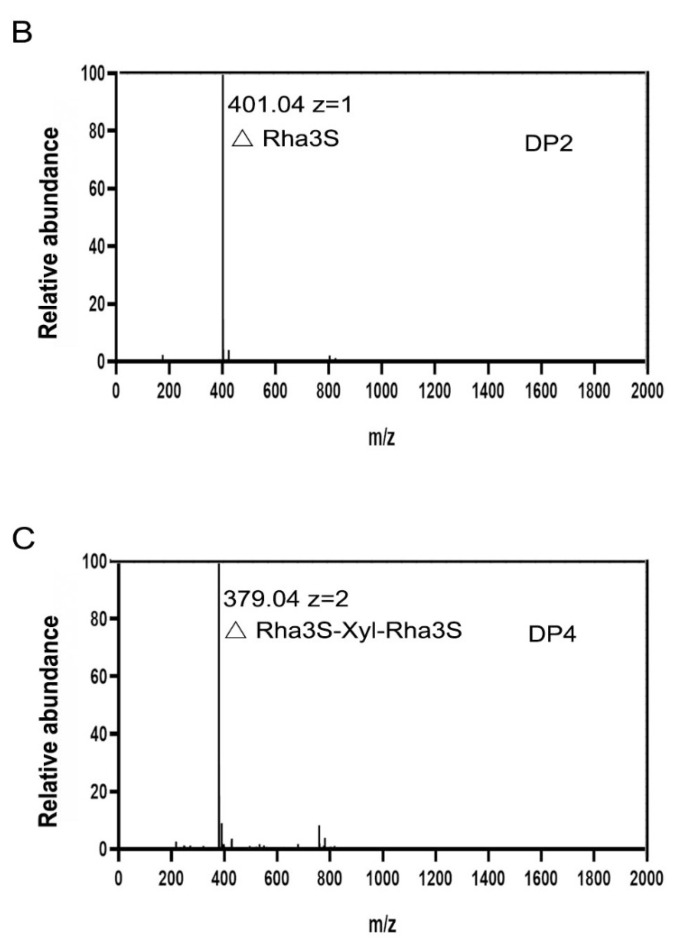
End products of TsUly25B. (**A**) The end product analysis using Superdex peptide 10/300 GL. (**B**,**C**) MS analysis of end products separated by Superdex peptide 10/300 GL. ∆ represents an unsaturated 4-deoxy-l-threo-hex-4-enopyranosiduronic acid. DP represents the degree of polysaccharides.

For the research about the mode of action, the degradation products collected at different time points were monitored by SEC. Through the result, an endolytic mode can be observed due to the phenomenon that ulvan oligosaccharides of high degrees of polymerizations (DPs) decreased as the reaction went on and the ones of low DPs increased (Figure 6A,B). Moreover, unsaturated ulvan disaccharide (∆Rha3S) and tetrasaccharide (∆Rha3S-Xyl-Rha3S) were confirmed to be major end products (Figure 7A,C), which ∆ represented an unsaturated 4-deoxy-l-threo-hex-4-enopyranosiduronic acid.

## 3. Discussion

Several studies have documented that ulvan oligosaccharides exert good activity in the physiological process. For example, E. Abouraïcha et al. found that ulvan oligosaccharides displayed more efficient antibacterial effects than ulvan polysaccharides in apple mildew [20]. Studies by Roberta Paulert et al. have shown that desulfated ulvan dimer can induce an oxidative burst in dicot cells to initiate a defense response against pathogens [21]. Huimin Qi et al. discovered that ulvan oligosaccharides with lower molecular weight behaved better in antioxidant reactions compared with the ones with higher molecular weight [22]. Therefore, there is considerable interest in degrading ulvan into oligosaccharides. Owing to the high salt concentration and easily available anions such as phosphate and sulfate, the marine environment is very different from land. Living in the sea, algae adapt to these conditions resulting in the covalent modifications of the polysaccharides. In addition, marine polysaccharides usually contain rare monosaccharide components such as iduronic acid in ulvan. As a result, the structure of algal polysaccharides is often more complex than the ones biosynthesized by land plants. Therefore, it is necessary to develop specific enzymatic tools to depolymerize green seaweed polysaccharides.

TsUly25B, a new ulvan lyase from PL25, was identified from *Thalassomonas* sp. LD5 in this study. TsUly25B contained five highly conserved PL25 amino acids including His^117^, His^137^, Tyr^182^, Arg^198^, and Tyr^240^ which had been proved to be crucial to catalytic reaction in PLSV_3936, the only structure-resolved PL25 ulvan lyase [30], and TsUly25B shared a highly similar structure with PLSV_3936. This result showed that TsUly25B may share a similar catalytic mechanism with PLSV_3936 towards ulvan. The properties of the enzyme were also studied. The maximum activity was shown at 60 °C and pH 9.0 in the presence of 500 mM NaCl and TsUly25B can also keep stable in a neutral or slightly alkaline environment. Compared with other characterized ulvan lyases, TsUly25B had the highest optimal temperature, pH, and salt tolerance. TsUly25B possessed a better pH tolerance than ALT3695 from *Alteromonas* sp. A321 [31], *Fa*PL28 from *Formosa agariphila* KMM 3901^T^ [32], and PsPL from *Pseudoalteromonas* sp. PLSV [36]. In detail, TsUly25B showed over 60% of activity in the pH 7.0–7.5 range in 50 mM Na_2_HPO_4_-citric acid buffer, while ALT3695 remained less than 50% of activity between pH 7.0 and 7.5. TsUly25B showed over 60% of activity in the pH 6.5–7.0 range in 50 mM Na_2_HPO_4_-NaH_2_PO_4_ buffer, while *Fa*PL28 was observed to have no more than 30% of activity in the same condition. TsUly25B showed over 60% of activity when the pH was over 9.0 in 50 mM Glycine-NaOH buffer, while PsPL preserved less than 40% of activity. Moreover, when incubated at 50 °C for 1 h without NaCl in 20 mM Na_2_HPO_4_-NaH_2_PO_4_ buffer, TsUly25B remained approximately 40% of activity and the addition of NaCl can advance its activity remaining. However, for *Fa*PL28, there was no activity observed over 40 °C in similar conditions. Two salt bridges between Arg^112^ and Asp^115^ were found near the conserved catalytic sites (within 4 Å) in the catalytic cavity of TsUly25B (Figure 2B). Unlike the ulvan lyases whose real structure had been resolved as well as enzymatic properties had been studied, no salt bridge has been discovered in the catalytic cavity of AsPL and Uly1 [5,33]. Extra energy provided by heating was required to break the two salt bridges to exert optimal activity. This may explain the reason why TsUly25B has the highest optimum temperature. Major disaccharides and minor tetrasaccharides were demonstrated to constitute the end products according to the SEC and ESI-MS results.

TsUly25B was salt-tolerant, and its thermal stability could be significantly enhanced by NaCl. TsUly25B preserved approximately 80% of activity after incubation in 20 mM Na_2_HPO_4_-NaH_2_PO_4_ buffer containing 3 M or 4 M NaCl for 48 h at 30 °C, while the activity of AsPL from *Alteromonas* sp. [5] decreased by 57.5% when being incubated in 20 mM Tris-HCl buffer containing more than 2.5 M NaCl after 24 h. The activity of *Fa*PL28 [32] was inhibited when the concentration of NaCl was over 200 mM in a similar condition. The salt dependence and adaptability may be related to the living environment of the source strain, since *Thalassomonas* sp. LD5 was screened from Qingdao seawaters. These properties make it more possible to store this protein at room temperature to save energy using NaCl as a stabilizing agent. In reviewing the literature, the phenomenon that proteins are salt-tolerant and sodium chloride can improve their stability is usually related to the hydrophilicity of surface amino acids. Increased surface acid charge and decreased hydrophobic amino acids can enhance the salt tolerance of enzymes. However, the electrostatic potential distribution confirmed that TsUly25B did not have a highly negatively charged surface (Figure 2C), which was different from AsPL [5] with highly negative electrostatic potential at the surface. In addition, the amino acid composition of TsUly25B is also not significantly different from other ulvan lyases with characterized structures which indicates that surface amino acids might not be the reason for the salt tolerance of TsUly25B. Sivakumar et al. proved that surface-exposed salt bridges can stabilize halophilic and thermophilic proteins AmyA which was a secretory α-amylase without an acidic surface [37]. This study may explain why TsUly25B has good salt tolerance for that there were 24 surface-exposed salt bridges detected by VMD. Further investigations on the real structure analysis are required to confirm and validate these supposes.

In the assay of enzymatic reaction kinetics of TsUly25, the turnover numbers (*k*_cat_) were calculated by the ratio of *V*_max_ versus enzyme concentration. The determination of Michaelis-Menten can be applied only to a Michaelian system. Neglecting enzyme inactivation can result in errors in both estimating the kinetics parameters and reporting the mechanisms of enzyme action. The most commonly used test for identifying enzyme inactivation is the “Selwyn test” [38,39]. Progress curves of the product formation were compared at several different initial enzyme concentrations. We found that plots of the product (A_235_) against time multiplied by the initial enzyme concentration were superimposable in 5 min. It implied that the concentration of active enzyme was varying and that product formation rates were dependent upon the change of enzyme concentration through time. For the enzymatic kinetics determination, the reaction time was 3 min. So, the *K*_m_ and *V*_max_ values can be investigated by the Michaelis–Menten equation.

TsUly25B can be a promising candidate for preparing oligosaccharides. TsUly25B showed a higher affinity for ulvan than PsPL which had a *K*_m_ of 2.10 mg·mL^−1^ [36]. The unsaturated ulvan disaccharide (∆Rha3S) was the major component (approximately 82%) in the enzymatic products. The whole reaction can be conducted at nearly room temperature, which means less energy consumption, indicating the potential for practical application.

## 4. Materials and Methods

### 4.1. Materials

The ulvan for the final enzymatic products study was extracted from dried seaweed *Ulva lactuca* using the hot water method [23]. Monosaccharide composition was elucidated by a 1-phenyl-3-methyl-5-pyrazolone (PMP)-High-Performance Liquid Chromatography (HPLC) method as described in Appendix A. The uronic acid content of the ulvan was described in Appendix B. The ulvan used for other experiments was from Elicityl-Oligotech (Crolles, France). The strain *Thalassomonas* sp. LD5 capable of degrading agar [40,41], alginate [42,43], and ulvan, was screened from Qingdao offshore. Restriction enzymes pET-28a (+) vector plasmid (from Takara Co., Ltd., Dalian, China), T4 ligase, restriction endonuclease *Nde* I and *Xho* I, and DNA polymerase (from Vazyme Biotech Co., Ltd., Nanjing, China) were used for Vector construction. *Escherichia coli* DH5α from TaKaRa (Dalian, China) was used for DNA cloning and *E. coli* BL21 (DE3) from TaKaRa (Dalian, China) was used for recombinant protein expression. The protein purification was conducted on the ÄKTA Fast Protein Liquid Chromatography (FPLC, Pittsburgh, PA, USA) equipping a Histrap HP column (5 mL, for the first step) and a Hitrap Q HP column (1 mL, for the second step). Histrap HP column (5 mL) and Hitrap Q HPcolumn (1 mL) from GE Healthcare were used for recombinant protein purification. Superdex peptide 10/300 GL from GE Healthcare was used for degradation product analyses. For protein concentration measurement, BCA Protein Quantification Kits (from Vazyme Biotech Co., Ltd., Nanjing, China) were used. Primer synthesis and gene sequencing were accomplished by the Beijing RuiBotech.

### 4.2. Sequence Analysis and Homology Modeling of TsUly25B 

Genome sequencing of *Thalassomonas* sp. LD5 was accomplished by the Beijing RuiBotech. Rast server (https://rast.nmpdr.org/rast.cgi, accessed on 5 October 2020) [44,45,46] and dbCAN meta server (https://bcb.unl.edu/dbCAN2/, accessed on 7 October 2020) [47,48] were used to analyze the genome of *Thalassomonas* sp. LD5 and find gene *tsuly*25B. SignalP 5.0 server (http://www.cbs.dtu.dk/services/SignalP/, accessed on 10 October 2020) was used for signal peptide prediction. Compute pI/ Mw tool (https://web.expasy.org/compute_pi/, accessed on 10 October 2020) was used for pI/Mw calculation. MEGA 7.0 was used for phylogenetic tree construction via the neighbor-joining method. SWISS-MODEL (https://swissmodel.expasy.org/, accessed on 10 October 2020) was used for three-dimensional structure modeling. PyMOL and VMD were used for salt bridges prediction. ClustalW and ESPript 3.0 (http://espript.ibcp.fr/ESPript/cgi-bin/ESPript.cgi, accessed on 10 October 2020) were used for amino acid sequence alignment. The three-dimensional structure of TsUly25B was modeled using PLSV_3936 (PDB ID: 5UAM) as the template. The sequence of TsUly25B was submitted to GenBank under accession number OK483196. Standard parameters were used for bioinformatics tools.

### 4.3. Cloning, Expression, and Purification of Recombinant TsUly25B

The gene *tsuly25B* was cloned by PCR using the genomic DNA of *Thalassomonas* sp. LD5 as the template (primer F: GGGAATTCCATATGGTTTAATTTTGACTTGCGCACTT, primer R: CCGCTCGAGTTTAAGTTGATAACGAGCCTTG). Then the PCR product was ligated to pET-28a (+) between *Nde* I and *Xho* I to acquire recombinant vectors. For the recombinant work, T4 ligase was used for linking linear pET-28a (+) and PCR products which were both cut using restriction endonuclease *Nde* I and *Xho* I. The constructed vectors were transformed into *E. coli* BL21 (DE3). The resulting cells during the logarithmic growth phase were induced by 0.25 mM isopropyl-*β*-d-thiogalactopyranoside (IPTG) at 18 °C and 160 rpm for 48 h in LB medium. After being collected at 4 °C and 12,000 rpm for 30 min, the cells were resuspended in 20 mM Na_2_HPO_4_-NaH_2_PO_4_ buffer (phosphate buffer, PB) (pH 8.0) with 500 mM NaCl. Then the cells were crushed by a high-pressure cell homogenizer (Juneng Nano & Bio Technology Co., Ltd., Guangzhou, China). The supernatant was collected using 12,000 rpm for 20 min, and the protein was purified by the two-step column chromatography with a flow rate of 1 mL/min. Different proteins with different affinity to stuffing of column can be separated by altering the percentage of elution buffer (0%, 5%, 20%, 40%, 60%, 100%). In the first step, the 6-his tag existing in pET-28a (+) was employed to aid purification. A total of 20 mM PB (pH 8.0) with 500 mM NaCl was used to balance the column and 20 mM PB (pH 8.0) with 500 mM NaCl and 500 mM imidazole was used as elution buffer. Target protein can be eluted when the percentage of elution buffer was 100%. In the second step, differences in protein charge were used for purification. Then, 20 mM PB (pH 8.0) with 0 M NaCl was used to balance the column, and 20 mM PB (pH 8.0) with 2 M NaCl was used as elution buffer. Target protein can be eluted when the percentage of elution buffer was 40%. Then the purified protein was desalted with dialysis. The tests of purity and molecular weight were performed on 10% (*w*/*v*) sodium dodecyl sulfate-polyacrylamide gel electrophoresis (SDS-PAGE).

### 4.4. Enzymatic Activity Assay

To make the substrate solution, ulvan was added into 20 mM PB (pH 9.0, 500 mM NaCl) to a final concentration of 0.1% (*w*/*v*). A total of, 0.1 mL of purified enzyme solution was added into 0.9 mL of substrate solution to constitute the reaction system. The reaction time was 10 min, and the reaction temperature was 60 °C. The ulvan lyase activity of TsUly25B was assayed by detecting the increase of the absorbance at 235 nm (A_235_) using a UV spectrophotometer (UH5300, Hitachi, Tokyo, Japan). Then, 100 μL of the completed deactivated enzyme was treated in the same way to make the corresponding blank. The enzyme was deactivated at 100 °C for 10 min. The amount of the protein required to release 1 μmol of unsaturated ulvan products with an extinction coefficient of 4800 M^−1^ cm^−1^ per minute was regarded as one unit (U).

### 4.5. Biochemical Characterization of Recombinant TsUly25B

The activity of recombinant TsUly25B was assayed at the temperature range from 0 °C to 80 °C to determine the optimal temperature. A total of 0.1% (*w*/*v*) ulvan was added into 20 mM PB (pH 9.0, 500 mM NaCl) to make the substrate solution. Then, 0.1 mL of purified enzyme solution was added into 0.9 mL of substrate solution. The reaction time was 10 min, and the reaction temperature was at 0 °C, 10 °C, 20 °C, 30 °C, 40 °C, 50 °C, 60 °C, 70 °C, and 80 °C, respectively. As to the influence of temperature on stability, recombinant TsUly25B was placed at various temperatures between 0 °C and 60 °C (0 °C, 10 °C, 20 °C, 30 °C, 40 °C, 50 °C, 60 °C) for 1 h before being tested residual activity at 60 °C using the same substrate solution and reaction time. 

The study to probe into the influence of pH on enzyme activity was carried out in different buffers with diverse pH. To obtain the experimental buffers with different pH, 50 mM Na_2_HPO_4_ citric acid (500 mM NaCl, pH 3.0–8.0), 50 mM Na_2_HPO_4_-NaH_2_PO_4_ (500 mM NaCl, pH 6.0–8.0), 50 mM Tris-HCl (500 mM NaCl, pH 7.05–8.95) and 50 mM Glycine-NaOH (500 mM NaCl, pH 8.6–10.6) were prepared. For optimal pH, 0.1% (*w*/*v*) ulvan was added into the above buffers, respectively, to make a substrate solution. A total of 0.1 mL of purified enzyme solution was added into 0.9 mL of substrate solution. The reaction time was 10 min, and the reaction temperature was 60 °C. For pH stability, TsUly25B was incubated in the above buffers respectively for 12 h at 4 °C and then the remaining activity was tested. Then, 0.1% (*w*/*v*) ulvan was added into 20 mM PB (pH 9.0, 500 mM NaCl) to make the substrate solution. Another 0.1 mL of purified enzyme solution was added into 0.9 mL of substrate solution. The reaction time was 10 min, and the reaction temperature was 60 °C.

The investigation of the optimal NaCl concentration for enzyme activity was executed using different substrate solutions in presence of various NaCl concentrations between 0 M and 1 M. As for NaCl stability, the protein was added into 20 mM PB containing a range of NaCl concentration at 0 °C, 30 °C, 40 °C and 50 °C for a series of time. Then the remaining activity was confirmed at optimal temperature.

As to the calculation of relative activity, for the determination of optimal temperature, pH, and concentration of NaCl, the activity at each condition was tested firstly and the highest activity obtained was set as 100%. Then the ratio of the activity of each point to the highest activity was calculated to get the relative activity. For the determination of stability, the residual activity after different treatments was tested firstly and the initial activity at each condition was set as 100%. Then the ratio was calculated.

### 4.6. Enzymatic Kinetics of Recombinant TsUly25B

Ulvan was dissolved in 20 mM PB (pH 9.0) with 0.5 M NaCl to reach a final concentration between 0.05 and 3 mg/mL. A total of 100 μL of purified TsUly25B was mixed with 900 μL of the substrate solution, then the mixture was placed at 60 °C for 3 min. A_235_ of the reaction mixture was measured. *K*_m_ and *V*_max_ values were investigated by the Michaelis–Menten equation and the curve fitting program by non-linear regression analysis using Graphpad Prism 8.

### 4.7. Action Mode and End Products of Recombinant TsUly25B

To determine the action mode, size exclusion chromatography (SEC) was employed to assay the enzymatic products at different time points (5, 10, 30, and 60 min) during the reaction process. The substrate solution was made by dissolving ulvan (1 mg/mL) into 20 mM PB (pH 9.0, 0.5 M NaCl). A total of 0.1 mL of purified TsUly25B (0.1 U/mL) was added into 0.9 mL of the substrate and this reaction system was placed at 30 °C. Superdex peptide 10/300 GL column was used to separate the products with 0.2 M NH_4_HCO_3_ as the mobile phase and a flow rate of 0.2 mL/min.

To obtain and analyze the end product, the following procedures were accomplished. Ulvan (10 mg) was dissolved in 1 mL PB (20 mM PB, pH 9.0, 0.5 M NaCl) to make the substrate solution. Purified TsUly25B (0.5 U once) was added into the substrate solution at 0 h, 2 h, 4 h, 6h at 30 °C and the total reaction time was 24 h. For TLC analysis, the mobile phase consisted of N-butanol, methanoic acid, and H_2_O (4:6:1), and the staining reagent consisted of acetone, phosphoric acid (85%), aniline, diphenylamine, and concentrated hydrochloric acid (246 mL: 20 mL: 4 mL: 4 g: 2mL). Superdex peptide 10/300 GL column was used to separate and purify the end products. The purified products were mixed completely in advance at a ratio of 1:1 (*v*/*v*) with acetonitrile, and the mixture was assayed using electrospray ionization-mass spectrometry (ESI-MS). The ESI-MS was in the negative ion mode on LTQ ORBITRAP XL (Thermo Fisher Scientific, Waltham, MA, USA) and parameters set containing tube lens, capillary voltage, capillary temperature, I spray voltage, sheath gas flow rate, and mass acquisition range was set at 35 V, 16 V, 275 °C, 2.5 kV, 10 arb and 100–2000, respectively.

## 5. Conclusions

In this study, a new PL25 ulvan lyase TsUly25B from *Thalassomonas* sp. LD5 was cloned, expressed, and characterized. TsUly25B possessed the highest optimal temperature and salt tolerance among all the characterized ulvan lyases. TsUly25B exhibited good thermal stability and sodium chloride can improve its thermal stability. This study also made reasonable preliminary analyses and predictions for structural information of TsUly25B using bioinformatics tools. Overall, due to the good characteristics, TsUly25B can be used as a tool enzyme to degrade ulvan to prepare ulvan oligosaccharides, and it can also be applied for further research on the structure–function relationship of ulvan lyases.

## Figures and Tables

**Figure A1:**
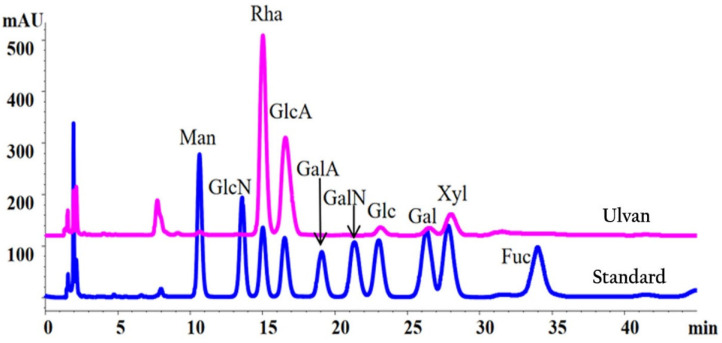
Monosaccharide composition of ulvan from *Ulva lactuca*. The pink line represents the analysis result of ulvan and the blue line represents the analysis result of the standard sample. The Man represents mannose. The GlcN represents glucosamine. The Rha represents rhamnose. The GlcA represents glucuronic acid. The GalA represents galactonic acid. The GalN represents galactosamine. The Glc represents glucose. The Gal represents galactose. The Xyl represents xylose. The Fuc represents fructose.

## Data Availability

Data is contained within the article.

## Appendix A

Analysis of monosaccharide composition of ulvan from *Ulva lactuca*. 1. Materials and methods: Monosaccharide composition was elucidated by a 1-phenyl-3-methyl-5-pyrazolone (PMP)-High-Performance Liquid Chromatography (HPLC) method [49,50]. Briefly, the ulvan sample (1 mg) in ampoule was dissolved into 4 M trifluoroacetic acid solution (200 μL), then sealed and hydrolyzed at 110 °C for 6 h. The obtained ulvan hydrolysate or monosaccharide standards (1 mg/mL) were derived by 1-phenyl-3-methyl-5-pyrazolone (PMP) at 70 °C for 1 h. The PMP-derived samples were analyzed on the HPLC system equipped with an Agilent Eclipse XDB-C18 column (150 mm × 4.6 mm, 5 μm) and a variable wavelength detector (245 nm). Monosaccharides were analyzed by using a mobile phase composed of 0.1 M KH_2_PO_4_ (pH 6.7)/acetonitrile (84:16, *v*/*v*). A mixture of 10 monosaccharides purchased from Sigma-Aldrich Company (St. Louis, MI, USA) was used as an internal standard. For other detection parameters, the column temperature was set as 30 °C, the flow rate was set as 1 mL/min, the injection volume was 10 μL, the detector was a UV detector and the acquisition time was 45 min. 2. Results: As shown in Figure A1, ulvan from *Ulva lactuca* is mainly composed of rhamnose (Rha), glucuronic acid (GlcA), xylose (Xyl). They accounted for 56.8 mol%, 33.1 mol%, and 6.7 mol%, respectively. In addition, small amounts of glucose (Glc) and galactose (Gal) were also present.

## Appendix B

Assay of the uronic acid content of the ulvan from *Ulva lactuca*. 1. Materials and methods: The uronic acid content of the ulvan was determined by using the carbazole-sulfuric acid method, and glucuronic acid purchased from Sigma-Aldrich Company (USA) was used as standard. The principle is that uronic acid can be hydrolyzed into furfural derivatives in the presence of concentrated sulfuric acid, and then the furfural derivatives can act with carbazole to generate purple-red compounds. Briefly, ulvan sample (0.4 mg) was dissolved into 1 mL of pure water to make the sample solution. The standard glucuronic acid was dissolved into 1 mL pure water to make standard solutions with a series of concentration (0, 0.08, 0.16, 0.24, 0.32, 0.4 mg/mL). Sulfuric acid-borax reagent (300 μL, 0.9 g borax/ 10 mL pure water/ 90 mL concentrated sulfuric acid) was added into sample solution and standard solutions, then reacted at 100 °C for 10 min. After cooling to room temperature, 0.1% carbazole (10 μL, 0.25 g carbazole/250 mL ethanol) was added, then reacted at 100 °C for 10 min. The absorbance at 562 nm was tested after cooling to room temperature using an EnSpireTM Multimode Plate Reader (PerkinElmer, Singapore). A standard curve can be drawn with the concentration and absorbance of the standard solutions as the horizontal and vertical coordinates respectively, and then the standard curve can be used as a control to determine the uronic acid content of the sample solution. Borax, carbazole and concentrated sulfuric acid were purchased from SINOPHARM (Beijing, China). 2. Results: The uronic acid content of the ulvan was 29.56% and the amount of cleavable bonds was 1.52 mmol/g.
